# Assessing Fear Following Retrieval + Extinction Through Suppression of Baseline Reward Seeking vs. Freezing

**DOI:** 10.3389/fnbeh.2015.00355

**Published:** 2015-12-23

**Authors:** Jason Shumake, Marie H. Monfils

**Affiliations:** Department of Psychology, The University of Texas at AustinAustin, TX, USA

**Keywords:** fear conditioning, freezing, conditioned suppression, extinction, reconsolidation, retrieval + extinction

## Abstract

Freezing has become the predominant measure used in rodent studies of conditioned fear, but conditioned suppression of reward-seeking behavior may provide a measure that is more relevant to human anxiety disorders; that is, a measure of how fear interferes with the enjoyment of pleasurable activities. Previous work has found that an isolated presentation of a fear conditioned stimulus (CS) prior to extinction training (retrieval + extinction) results in a more robust and longer-lasting reduction in fear. The objective of this study was to assess whether the retrieval + extinction effect is evident using conditioned suppression of reward seeking, operationalized as a reduction in baseline licking (without prior water deprivation) for a 10% sucrose solution. We found that, compared to freezing, conditioned suppression of reward seeking was much more sensitive to fear conditioning and far less responsive to extinction training. As in previous work, we found that retrieval + extinction reduced post-extinction fear reinstatement when measured as freezing, but it did not reduce fear reinstatement when measured as conditioned suppression. This suggests that there is still residual fear following retrieval + extinction, or that this procedure only modifies memory traces in neural circuits relevant to the expression of freezing, but not to the suppression of reward seeking.

## Introduction

Freezing, or becoming motionless in the presence of fear-evoking stimuli, is one of the innate defensive reactions of rats and other rodents, and it has become the predominant measure—often the only behavioral measure—used in studies of conditioned fear/threat. However, historically this was not always the case. For several decades following its introduction by Estes and Skinner (1941), conditioned suppression was the predominant technique for measuring conditioned fear. In the prototypical conditioned suppression paradigm, food-deprived rats are first trained to press a lever to receive food reward. Then a conditioned stimulus (CS) is paired with a shock unconditioned stimulus (US). Subsequently, rats suppress lever responding when the CS is present, and the magnitude of this suppression offers an indirect measure of conditioned fear.

Eventually, the numerous advantages offered by conditioned freezing led to its dominant use in studies of Pavlovian fear conditioning. Namely, freezing behavior can be directly observed without the need for extensive prior operant training or the accompanying states of physiological deprivation required to elicit robust operant responding. Such a simplified preparation is especially appealing for uncovering the neurobiological mechanisms specific to fear learning because one does not need to control for the neural effects of altered motivational states or concurrent appetitive learning. However, in recent years, there has been increasing interest in translating findings from Pavlovian fear conditioning to the treatment of human anxiety disorders. For this objective, conditioned suppression may be more relevant than freezing to the way that human beings experience fear, i.e., as something that interferes with the enjoyment of pleasurable activities (McDannald and Galarce, 2011).

We recently reported that a modified extinction paradigm, retrieval + extinction (Ret + Ext), resulted in a persistent attenuation of fear memories, leaving them less susceptible to return of fear as evidenced by several measures, including resistance to fear reinstatement following unsignaled shock presentations (Monfils et al., 2009). This finding has been replicated both in rodents and humans (Schiller et al., 2010, 2013; Rao-Ruiz et al., 2011; Olshavsky et al., 2013a; for a review, Auber et al., 2013; but see also: Chan et al., 2010) and extended to appetitive memories (Xue et al., 2012; Olshavsky et al., 2013b; Sarter and Ashton-Jones, 2014). What is not yet known is whether the original fear memory is being erased (we do not believe this to be the case; Tedesco et al., 2014) or updated, and, if the latter, to what extent the CS is still perceived as a threat. This is impossible to assess using freezing alone because the absence of freezing does not necessarily indicate the absence of fear. As discussed by Blanchard and Blanchard (1988), animals do not shift abruptly from freezing to normal behavior (eating, drinking, aggression, and sexual activity); rather, there is a protracted intermediate period of risk assessment, characterized by cautious exploration and the *suppression of unnecessary activities*. Thus, the main objective of this study was to assess whether the Ret + Ext procedure is successful not only at preventing reinstatement of conditioned freezing, but also at preventing reinstatement of conditioned suppression.

We further introduce in this study a modified version of the conditioned suppression procedure to mitigate its major drawbacks: namely, the need for food or water deprivation and operant response training. We have previously shown that, when placed in an operant box with free access to water sweetened with sucrose, rats will voluntarily spend a substantial percentage of time drinking without the need for prior water deprivation (Shumake et al., 2005; Hamani et al., 2010). Moreover, licking behavior does not require special training and can be automatically and precisely quantified using an optical lickometer. While a deprivation period would no doubt result in more robust drinking behavior, our objective was to simulate conditions under which a human patient with an anxiety disorder might experience dysfunction, i.e., conditions which typically do not involve severe hunger or thirst. In other words, we wanted the response competing with fear to be motivated by pleasure, not survival.

Arguably “pleasure drinking” offers not only greater translational relevance to humans, but also a more sensitive instrument with which to measure fear itself. We submit that “absolute zero” on the fear-measurement scale should be operationalized as a complete return to normal behavior in a safe environment, and that neither 0 freezing nor 0 suppression of “survival drinking” offers sufficient evidence that this has occurred. For reasons already discussed, the CS can be perceived as threatening without evoking freezing. Likewise, eating or drinking under conditions of extreme hunger or thirst does not demonstrate that the CS is no longer threatening; rather, it only demonstrates that the threat of the CS is less severe than the threat of starvation or dehydration. Drinking for the simple pleasure of experiencing a sweet taste, on the other hand, is not a necessary activity. Therefore, if Ret + Ext behavior restored and preserved this behavior, it would offer stronger evidence that the original fear memory had been fundamentally rewritten. We tested this hypothesis by comparing the long-term memory of fear vs. extinction learning in animals who received either standard extinction or Ret + Ext, as assessed by freezing vs. suppression of drinking following acquisition, extinction, and reinstatement.

## Methods

### Animals

A total of 32 male Sprague-Dawley albino rats (Charles River Laboratories) arrived in our animal facility at approximately 50 days of age and were pair-housed (2 per cage). Rooms were maintained at steady temperature (21 ± 1°C) and a 12-12 light-dark cycle (lights on at 7:00 and off at 19:00). Except for one 24-h period of water deprivation as described below, food and water were provided *ad libitum*. All procedures followed US National Institutes of Health guidelines and were approved by the Institutional Animal Care and Use Committee at the University of Texas at Austin.

### Apparatus

Rats were conditioned and tested in a Habitest Modular System (Coulbourn Instruments) equipped with metal rod flooring connected to a shock generator, a speaker connected to a tone generator, and an optical lickometer that continuously monitored licking of an attached water bottle. Rats were videoed by overhead cameras. Graphic state software controlled stimulus presentations and recorded lickometer data. Raw data files from these sessions were exported as text files, and stimulus-dependent changes in licking behavior were quantified using a custom-written R package, “lickometer,” which can be downloaded from https://github.com/jashu/graphic-state-munging.

### Procedure

**Figure 1:**
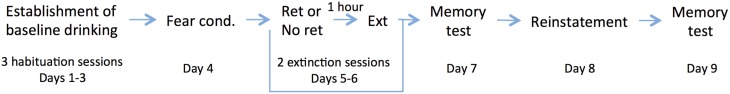
**Experimental timeline**.

A schematic of the experimental design is shown in Figure 1.

#### Establishment of baseline drinking (Days 1–3)

**Figure 2:**
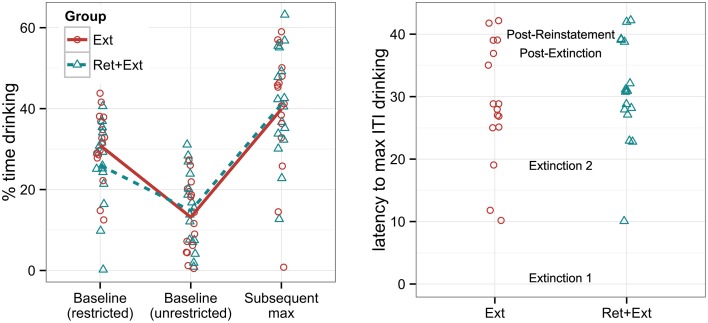
**Left panel:** Percent time that rats spent drinking a 10% sucrose solution under baseline conditions with and without water restrictions, labeled “Baseline (restricted)” and “Baseline (unrestricted),” respectively, and the maximum ITI drinking reached either during or following extinction, labeled “Subsequent max.” **Right panel:** The latency (given by the cumulative number of tone-alone presentations across experimental sessions) for each individual to reach its personal maximum ITI drinking (the “Subsequent max” measurement given in the left panel). The session labels in the middle of the graph indicate when each session began with respect to the y-axis. Note that group assignment was not made until after the baseline drinking measures were collected, and groups were explicitly matched for baseline drinking behavior. There were no significant group differences in either the maximum drinking rate or in the latency to reach it.

Following 1 week of acclimation after arrival at our facility, rats underwent 3 daily sessions of habituation to the conditioning chamber to establish a baseline rate of drinking (Figure 2), each session consisting of 10 min in the conditioning chamber with access to a bottle of drinking water with a 10% concentration of sucrose. Based on pilot data in which we ran subjects under conditions of both restricted and unrestricted access to water before assessing baseline drinking, we found that most rats (80%) that were unrestricted reached levels of drinking comparable to the restricted rats after 4 days of habituation sessions (15 min per day). In order to shorten the required habituation period, we adopted the hybrid paradigm used in this study, in which rats were water-deprived 24 h prior to the first habituation session in order to motivate them to overcome neophobia for drinking in the novel chamber, but then the restriction condition was removed for all subsequent habituation, training, and testing sessions.

#### Fear conditioning, extinction, and reinstatement (Days 4–9)

**Figure 3:**
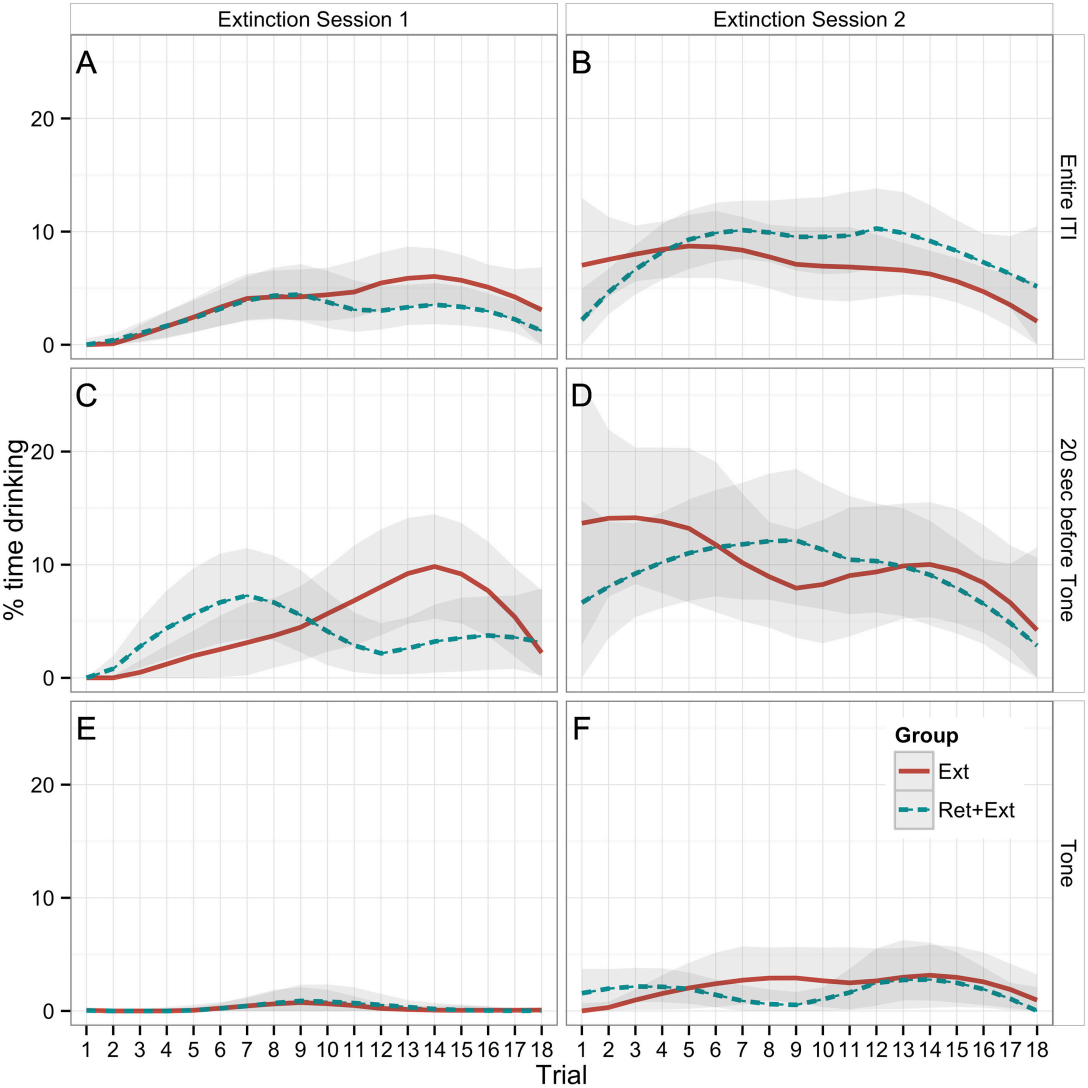
**Percent time that rats spent drinking during the first extinction session (A,C,E) and the second extinction session (B,D,F) across all 18 trials**. The **(A,B)** (Entire ITI) shows drinking as averaged across the entire ITI for each trial (the entire time between tones), and the **(C,D)** (20 sec before Tone) shows drinking during the 20 s immediately preceding each tone. Both can be used to compare with drinking during the 20 s Tone CS **(E,F)**. The Ret + Ext group experienced a 1-h break between the first and second trials of both extinction sessions (Extinction Sessions 1 and 2). Lines represent LOESS-predicted extinction curves with bootstrapped 95% confidence bands. Extinction to context was significantly greater than extinction to tone. There were no significant group differences.

On Day 4 (the day following the last habituation session), rats underwent fear conditioning. Rats received 3 conditioning trials of a tone (5 kHz for 20 s) co-terminating with a foot shock (0.7 mA for 0.5 s) separated by a variable intertrial interval (ITI) of 1–5 min. This was followed by 2 days of extinction (“Extinction Session 1” and “Extinction Session 2” in Figure 3) in which rats either received a standard extinction protocol (Ext) or a retrieval-plus-extinction protocol (Ret + Ext). Assignments to the Ext and Ret + Ext groups (*n* = 16 per group) were made based on cage-wise matching of baseline drinking behavior: after calculating the mean baseline drinking for each cage pair, cages were matched according to these means. This was done in order to assign cage mates to the same experimental condition while minimizing differences in baseline drinking motivation between the Ext and Ret + Ext groups.

**Figure 4:**
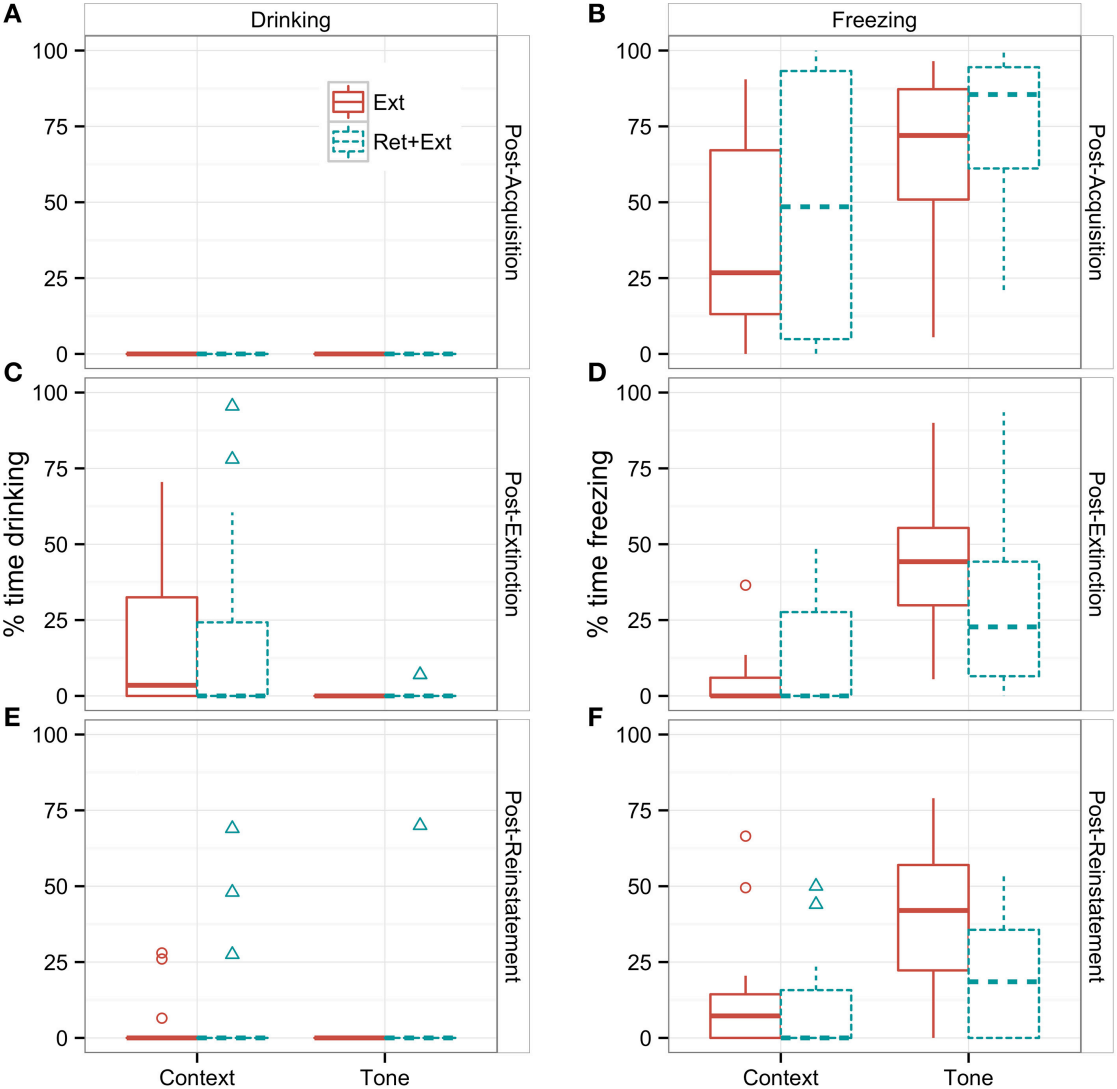
**Tests of fear vs. extinction memory as indicated by percent time drinking (A,C,E) and percent time freezing (B,D,F) measured during pre-CS (Context) vs. CS (Tone) during the first post-acquisition trial (A,B), post-extinction trial (C,D), and post-reinstatement trial (E,F)**. Data are presented as boxplots to demonstrate their range and distribution, which differ markedly between drinking vs. freezing and between tone vs. context. Boxes represent the middle 50% of the distribution (the interquartile range between the 25th and 75th percentiles), and the horizontal line indicates the median. The “whiskers” that extend vertically from the box indicate the range of observations that fall within ±1.5 times the interquartile range, and any observations outside the whiskers are graphed as individual points. Note the complete suppression of drinking **(A)** vs. the large variability in freezing **(B)** following fear conditioning. A significant mean difference between Ext vs. Ret + Ext groups was only observed for tone-CS freezing following reinstatement **(F)**.

On Days 5–6 (Extinction Session 1 and Extinction Session 2), rats were returned to the acquisition context for 4 min, during which time the Ret + Ext group (but not the Ext group) received a single 20 s tone. Rats were then returned to their home cages for 1 h and then reintroduced to the same context for extinction training, consisting of tone-alone presentations of the same duration and ITI as experienced in acquisition until both Ext and Ret + Ext groups had heard a total of 18 tones. On Day 7 (“Post-Extinction” in Figure 4), rats received 3 memory-recall trials of tone-alone presentations, again with the same duration and ITI parameters. On Day 8, rats received 2 unsignaled footshocks. On Day 9 (“Post-reinstatement” in Figure 4), they were tested for fear reinstatement, assessed by their freezing to the tone alone.

### Data analysis

#### Units of measurement

Since its advent by Annau and Kamin (1961), the conditioned suppression ratio (CS responding/CS responding + pre-CS responding) is traditionally used when reporting the results of a conditioned suppression experiment, but we are not using Kamin's ratio in this report. For one reason, there were many trials when there was no drinking during either the CS or pre-CS interval, which would result in division-by-zero errors if not modified. Moreover, such normalization is not conventionally performed for freezing measures, which are expressed in units of percent time. Therefore, we used percent-time units for reporting both drinking and freezing measures.

#### Analysis of drinking motivation

Paired *t*-tests were used to evaluate the effect of water restriction on drinking behavior during habituation to the test chamber, and to compare the water-restricted rate of drinking to the subsequent maximum rate of ITI drinking after extinction training had begun. In addition, we used independent *t*-tests to evaluate group differences in maximum drinking rate and the latency to reach it.

#### Analysis of extinction curves

Based on previous work, we did not expect the Ret + Ext manipulation to result in significant group differences *during* extinction learning, but, to be thorough, we included experimental group as an independent variable in these analyses. Extinction data were first analyzed with a 2 × 2 × 18 × 2 (Group × Session × Trial × CS) repeated measures ANOVA, with Group (Ext vs. Ret + Ext) as the between-subjects measure and Session, Trial, and CS as within-subject measures. Session was included to evaluate between-session extinction, operationalized as increased drinking between the first and second extinction sessions. Trial was included to evaluate within-session extinction, operationalized as increased drinking over the 18 trials. CS was included to evaluate the specificity of fear acquisition and extinction to the tone CS vs. contextual cues. For the above ANOVA, the effect of CS was operationalized as a difference between drinking during the tone vs. the 20 s preceding the tone, in order to match the CS and pre-CS in terms of temporal proximity and duration. However, one limitation of pleasure-motivated (as opposed to thirst-motivated) drinking is that it is more erratic, i.e., characterized by many spontaneous starts and stops. Thus, aggregating over longer intervals may provide a more reliable index of fear by averaging out this source of noise. Therefore, we also conducted a separate 2 × 2 × 18 (Group × Session × Trial) repeated measures ANOVA of contextual extinction alone, in which drinking was averaged over each ITI.

In addition, we used LOESS (LOcal regrESSion) to fit separate extinction curves (drinking as a function of trial number) within each Group × Session × CS cell. This nonparametric method constructs a nonlinear “smooth” of the drinking data over time, using local polynomial regression fitting (which requires no prior assumptions about the distribution of the data or the shape of the curve to be fit) to extract signal (systematic variation) from noise (random variation). Plots of these LOESS smooths provide an elegant way to visually compare differences in extinction curves. Moreover, confidence intervals can be constructed for the curve fits themselves, providing a gauge for when a curve significantly diverges from a reference point (e.g., 0) or from another curve without the need for multiple statistical tests at multiple time points.

#### Analysis of long-term memory (LTM)

Long-term changes in fear expression caused by fear conditioning, Ext vs. Ret + Ext, and reinstatement were assessed using data from the first tone presentation 24 h after the end of each of these training protocols. Separate but parallel ANOVAs were applied to the drinking and freezing data using a 2 × 3 × 2 design (Group × Session × CS), with group as a between-subject variable and session (post-acquisition vs. post-extinction vs. post-reisntatement) and CS (context vs. tone) as within-subject variables. Note that we also performed an analysis that included repeated measures of trial, which did not result in any substantive insights beyond the analysis of just the first trials. Moreover, our experimental manipulation was introduced following the first post-acquisition trial (meaning that the first trial provides the only pure baseline measure of fear acquisition), and the first trial of an LTM session following extinction or reinstatement training is the most likely to show spontaneous recovery or savings, respectively. For all these reasons, we chose to confine our analysis of LTM to the first trial.

#### Permutation and bootstrap *p*-values and confidence intervals

As can be seen in Figures 3, 4, fear conditioning suppressed drinking to floor levels. The severity of the floor effect varied as a function of training, time, and CS presentation, but nearly all time points showed distributions heavily skewed toward 0 with very long tails. Since this would appear to severely violate ANOVA assumptions, we did not rely on the theoretical sampling distribution of the *F* statistic to calculate *p*-values (i.e., the *p*-value output of standard analysis software); rather, we used random permutations to generate an empirical *F* distribution as described by Manly (2007). The observations were permuted (randomly reshuffled) 5000 times, simulating the distribution under which the null hypothesis would be true: under data randomization, any relationship between the independent and dependent variables is due to chance. Each permutation was performed in two stages to maintain the distinction of within- vs. between-subject variance: first, observations were permuted within each subject, and then group assignments were permuted between subjects. The ANOVAs outlined above were recomputed for each permutation, and the *F* statistic was recorded for each of the main effects and interactions. Thus, for each effect, we obtained a sampling distribution of the *F*-values that would be expected under the null hypothesis for such an unusually distributed measure. The *p*-value is then approximately equivalent to the proportion of *F*-values from this sampling distribution that are more extreme than the one obtained from our original data. The exact formula is (*r* + 1)/(*n* + 1), where *r* is the number of permutations that resulted in an *F* statistic greater than or equal to the original *F* statistic, and *n* is the total number of permutations.

Although the freezing data were not characterized by such severely skewed distributions, for consistency, we calculated empirical *p*-values for these data as well. Both theoretical and empirical *p*-values are given in the ANOVA summary tables (Tables 1–**4**). In the case of significant interactions involving group, simple effects of group were calculated using analogous permutation tests for group-mean differences within each level of the interacting variables (**Table 5**). Likewise, for consistency, we calculated the 95% confidence intervals surrounding the LOESS curves in Figure 3 using a bootstrap procedure in which the data for each curve were randomly resampled 5000 times with replacement and a LOESS curve was fit to each resample. The confidence intervals in Figure 3 correspond to the 2.5–97.5 percentile range of these 5000 different fits.

**Table 1:** **ANOVA of CS vs. pre-CS drinking time (fear of tone vs. context) during extinction learning**.

**Effect**	***df***	***F***	**Theoretical *p***	**Empirical *p***
Group	1, 30	0.4	0.53	0.54
**Session**	**1, 30**	**41.4**	<**0.001**	<**0.001**
Trial	17, 510	1.3	0.22	0.22
**CS**	**1, 30**	**76.8**	<**0.001**	<**0.001**
Group × Session	1, 30	0.2	0.69	0.68
Group × Trial	17, 510	0.8	0.64	0.64
Group × CS	1, 30	0.2	0.65	0.64
Session × Trial	17, 510	0.7	0.80	0.79
**Session** × **CS**	**1, 30**	**14.8**	<**0.001**	<**0.001**
Trial × CS	17, 510	1.2	0.26	0.27
Group × Session × Trial	17, 510	0.9	0.53	0.51
Group × Session × CS	1, 30	0.004	0.95	0.94
Group × Trial × CS	17, 510	0.8	0.66	0.66
Session × Trial × CS	17, 510	1.1	0.35	0.34
Group × Session × Trial × CS	17, 510	0.8	0.68	0.69

*Statistically significant effects are emphasized in boldface*.

#### Software

Figures were generated using the ggplot2 package for R (Wickham, 2009). Data were analyzed in RStudio (Version 0.99.467) using R (Version 3.2.2).

## Results

As described in the methods, the distribution of the drinking data severely violated ANOVA assumptions underlying the theoretical distribution of the *F* statistic used to calculate *p*-values. Therefore, we generated an empirical distribution of the *F* statistic using unrestricted permutation of the raw data within subjects and permutation of the group labels (Ext vs. Ret + Ext) between subjects. For consistency across analyses, we did the same for the freezing data as well. Both types of *p*-values, theoretical and empirical, are given in Tables 1–**5**. As expected, the two methods yielded highly consistent *p*-value estimates for the freezing data, to within rounding error of the hundredth decimal place. To our surprise, the two methods were also highly consistent for the drinking data. We caution that this result should not be taken as evidence that one can always ignore the presence of many zero values, which have the potential to exaggerate or diminish statistical effects depending on the proportion of zeros in each group and the direction of mean differences (see Delucchi and Bostrom, 2004, for a review of this problem and recommendations for analysis).

### Baseline drinking

Figure 2 shows that baseline rates of drinking were considerably higher under water-restricted vs. unrestricted conditions, *t*_(31)_ = 6.5, *p* < 0.001. However, approximately 60% of rats were drinking at a baseline rate within the range shown by rats after water deprivation (not counting one rat that showed zero drinking under water restriction). Figure 2 also shows that rats achieved even higher rates of drinking following extinction (typically during the second extinction session), reaching a maximum ITI drinking rate that was significantly greater than even the rate observed under water restriction, *t*_(31)_ = 4.9, *p* < 0.001. Recall that water restriction was implemented only during the first exposure to the apparatus, so this result does not reflect an effect of water restriction so much as an effect of extensive habituation to the apparatus; in other words, we do not know whether water deprivation would have resulted in higher maximum drinking rates had it been implemented for the entire experiment. However, the important point here is that most rats were spontaneously motivated to drink a sweetened solution at a substantial rate without the need for water restriction. Note that there was no significant group difference in the maximum drinking rate using a Welch two sample *t*-test, *t*_(29.4)_ = 0.2, *p* = 0.82, and no significant group difference in the latency to reach maximum drinking, *t*_(29.3)_ = 0.6, *p* = 0.58.

### Drinking during extinction

Table 1 reports the results of a Group × Session × Trial × CS ANOVA of drinking behavior within and between the 2 extinction sessions. The main finding was a significant Session × CS interaction (*p* < 0.001), indicating that drinking during the 20 s preceding the CS showed a significantly greater between-session increase than did drinking during the tone CS. Plots of extinction curves show that while there was a slight between-session increase in drinking during the tone CS (Figures 3E,F), tone-CS drinking remained greatly suppressed relative to the drinking observed during the 20 s before the tone CS (Figures 3C,D).

Table 2 reports the results of a Group × Session × Trial ANOVA of drinking during the entire ITI (Figures 3A,B), which, as detailed in the Methods, provides a less noisy (lower within-subject variance) index of contextual fear. In addition to the significant effect of Session, this analysis revealed a significant effect of Trial (*p* < 0.001). Plots of extinction curves show a gradual increase from an initially complete suppression of drinking during the first extinction session (Figure 3A). Rats began the second extinction session (Figure 3B) with an even higher rate of ITI drinking that remained steady for most of the session but declined toward the end, presumably because of satiety.

**Table 2:** **ANOVA of ITI drinking time (fear of context alone) during extinction learning**.

**Effect**	***df***	***F***	**Theoretical *p***	**Empirical *p***
Group	1, 30	0.05	0.82	0.82
**Session**	**1, 30**	**42.2**	<**0.001**	<**0.001**
**Trial**	**17, 510**	**3.0**	<**0.001**	<**0.001**
Group × Session	1, 30	2.9	0.10	0.09
Group × Trial	17, 510	1.2	0.27	0.26
Session × Trial	17, 510	1.2	0.23	0.22
Group × Session × Trial	17, 510	1.2	0.24	0.23

*Statistically significant effects are emphasized in boldface*.

In summary, the results indicate that pleasure-motivated drinking shows CS specificity and responds to extinction training. The Ret + Ext manipulation was not hypothesized to cause behavioral changes during extinction training, and no significant effects of Group were found (Tables 1, 2).

### Long-term memory as assessed by freezing vs. drinking

As the top row of Figures 4A,B illustrates, fear memory 24 h after acquisition looks very different when viewed through the lens of freezing vs. drinking behavior. There were large individual differences in conditioned freezing. The majority of rats showed high levels of freezing to tone, but several rats showed low levels of freezing. However, as the drinking data illustrate, low-freezing rats did acquire an aversive association to the tone: not one out of the 32 rats spent even a brief moment drinking during the post-acquisition trial. All rats showed a complete suppression of drinking.

Table 3 reports the results of the Group × Session × CS ANOVA of drinking during the memory trials. There was no significant effect of Ret + Ext on conditioned suppression. As with the above analysis of the full extinction sessions, the main finding was a significant Session × CS interaction (*p* = 0.004), indicating that the relative difference in drinking time during tone vs. context changes as a function of training. Both tone and context showed complete conditioned suppression following acquisition (Figure 4A), but only context showed extinction of conditioned suppression (Figure 4C). Following reinstatement training, complete conditioned suppression to context returned for all but 3 subjects per group (Figure 4E).

**Table 3:** **ANOVA of drinking time during memory trials**.

**Effect**	***df***	***F***	**Theoretical *p***	**Empirical *p***
Group	1, 30	1	0.33	0.32
**Session**	**2, 60**	**6.8**	**0.002**	**0.001**
**CS**	**1, 30**	**14.6**	<**0.001**	<**0.001**
Group × Session	2, 60	0.5	0.58	0.59
Group × CS	1, 30	0.06	0.81	0.81
**Session** × **CS**	**2, 60**	**5.5**	**0.006**	**0.004**
Group × Session × CS	2, 60	0.01	0.99	0.99

*Statistically significant effects are emphasized in boldface*.

Table 4 reports the results of the same ANOVA design for freezing during the same trials. This ANOVA revealed that Ret + Ext training caused significant changes in freezing behavior, as evidenced by a Group × Session interaction (*p* < 0.05). The Ret + Ext group showed a greater reduction in freezing between post-acquisition (Figure 4B) and post-extinction (Figure 4D) and post-reinstatement (Figure 4F). This effect was specific to the tone CS, as evidenced by a significant Group × CS interaction (*p* < 0.01). Simple effects tests of the interactions (Table 5) showed that a significant group difference (*p* < 0.05) was only evident for the tone CS following reinstatement. Note that, based on previous findings, this was the only condition in this experiment under which the Ret + Ext group was hypothesized to show less post-reinstatement freezing.

**Table 4:** **ANOVA of freezing time during memory trials**.

**Effect**	***df***	***F***	**Theoretical *p***	**Empirical *p***
Group	1, 30	0.02	0.89	0.90
**Session**	**2, 60**	**46.4**	<**0.001**	<**0.001**
**CS**	**1, 30**	**109.9**	<**0.001**	<**0.001**
**Group** × **Session**	**2, 60**	**3.2**	**0.05**	**0.04**
**Group** × **CS**	**1, 30**	**8**	**0.008**	**0.005**
Session × CS	2, 60	1.3	0.29	0.30
Group × Session × CS	2, 60	1.7	0.18	0.18

*Statistically significant effects are emphasized in boldface*.

**Table 5:** **Simple effects of group differences in freezing to context vs. CS for each memory test**.

**Session**	**CS**	***t***	**Theoretical *p***	**Empirical *p***
Post-Acquisition	Context	−0.8	0.42	0.41
	Tone	−1	0.33	0.32
Post-Extinction	Context	−1.7	0.1	0.1
	Tone	1.3	0.19	0.2
**Post-Reinstatement**	Context	0.52	0.61	0.62
	**Tone**	**2.4**	**0.02**	**0.02**

*Statistically significant effects are emphasized in boldface*.

## Discussion

We previously reported that an isolated presentation of a fear CS prior to extinction training (retrieval + extinction) results in a more robust and longer-lasting reduction in fear as measured by conditioned freezing after reinstatement. In the present study, we assessed whether the retrieval + extinction effect is evident when conditioned suppression is used to measure fear. Freezing has become the predominant measure used in rodent studies of conditioned fear, but conditioned suppression may provide a measure that is more relevant to human anxiety disorders; that is, a measure of how fear interferes with the enjoyment of pleasurable activities. As in previous work, we found that retrieval + extinction reduced fear reinstatement after extinction when measured as freezing, but it did not reduce fear reinstatement when measured as conditioned suppression. Our results suggest that there is still residual fear following retrieval + extinction, or that this procedure is only modifying memory traces in neural circuits relevant to the expression of freezing but not the expression of conditioned suppression.

Bouton and Bolles (1980) reported that freezing was reliably correlated with the suppression of several consummatory behaviors, including licking for a sucrose solution as used in our study. However, their study deprived rats of food and water for 48 h prior to testing, and, to our knowledge, all studies utilizing the conditioned suppression paradigm have similarly used food or water restriction to instill an intense consummatory drive to compete with conditioned fear. In studies of fear extinction, this may lead to the false impression that when rats resume consummatory behaviors, they are no longer experiencing fear. But an alternative possibility is that the severe physiological challenge of food or water deprivation creates a survival emergency. Interpreted in this way, rats that resume drinking water in the presence of a conditioned fear stimulus have determined that the threat to their survival from dehydration outweighs the threat imposed by the CS; it implies that the CS has becomes less threatening, but it does not necessarily imply that the CS is no longer perceived as a threat. There may be substantial residual fear—not enough to cause freezing, but enough to suppress the pursuit of rewards for the sake of pleasure as opposed to the sake of survival.

Our results show that, not surprisingly, rats consume sweetened water at a higher rate when they have been water deprived, but most were still motivated to drink without prior deprivation because presumably the sweet solution was rewarding in itself. When the survival imperative associated with thirst was no longer a factor, we observed a sharp dissociation between freezing levels and drinking behavior. Whereas fear acquisition levels in terms of freezing were highly variable, acquisition of conditioned suppression was both absolute and invariant. Two extinction sessions were required before some rats began to drink during the tone CS. However, most responded to the extinction session by drinking substantially during the ITI and drinking minimally during the CS. This behavior may have more translational relevance to fear-related psychopathology, reflecting that fear-associated stimuli can continue to elicit sufficient wariness and vigilance to disrupt normal life. The complete extinction of these fear memories would seem to pose a much greater challenge than the extinction of freezing, which may represent only a diminution of fear.

To this end, we were interested to see if the Ret + Ext paradigm, which has been proposed to lead to a disruption in the reconsolidation of fear memories (Monfils et al., 2009), might be successful in normalizing drinking behavior following conditioned suppression. While we replicated previous work finding that Ret + Ext training inhibits the reinstatement of fear as measured by freezing, there was no evidence that Ret + Ext made any difference in the expression of fear as measured by conditioned suppression. One limitation of the drinking measure is that any given animal on any given trial may take a break from drinking that is unrelated to the presence or absence of the CS. (This is indeed the impetus for prior deprivation, which increases the likelihood of continuous drinking.) However, with a sufficient number of subjects, one should still be able to detect the effects of experimental manipulations from the aggregate data. Indeed, the extinction curves reveal an obvious difference between measures of drinking during the 20 s prior to the CS (Figures 3C,D) vs. the 20 s during the CS (Figures 3E,F).

There are several ways to interpret this finding in terms of the effect of the Ret + Ext paradigm on memory mechanisms. First, it seems clear that the fear memory is not “erased” in its entirety, but it is still possible that the memory is weakened beyond what is achieved by Ext alone. In this view, the fear memory is weakened enough to prevent reinstatement of freezing for the average animal, but not so much as to remove the wariness of drinking during the CS. Another possibility is that fear conditioning instantiates multiple memory traces in parallel neural circuits, only some of which may be vulnerable to disruption by the Ret + Ext manipulation. For example, despite its prominent and well-established role in conditioned freezing, the basolateral amygdala (BLA) has been reported to play a minimal role in conditioned suppression (Killcross et al., 1997; Lee et al., 2005; Petrovich et al., 2009; McDannald and Galarce, 2011). Thus, to the extent that the Ret + Ext manipulation selectively targets BLA neuroplasticity, we would expect it to have a greater impact on conditioned freezing than on conditioned suppression.

Finally, Figure 4F illustrates that Ret + Ext shifted the distribution of freezing scores toward floor levels of freezing, but there was still substantial overlap with standard extinction. In other words, for some individuals, two sessions of standard Ext training were sufficient to prevent reinstatement of conditioned freezing while, for others, Ret + Ext was not enough. Thus, it seems likely that individual differences moderate the response to extinction paradigms, and it will be important for future research efforts to uncover the relevant phenotypes (Olshavsky et al., 2013a).

Moreover, when animals are drinking to experience the pleasure of a sweet taste (as opposed to quenching an experimentally induced thirst), fear conditioning appears to cause far more indelible behavioral changes. All of the rats resumed drinking by the end of the second extinction session, but they confined their drinking almost entirely to the time between tones (Figure 4, right column). Very few were willing to continue drinking in the presence of the tone CS. This persistent wariness may have far more clinical relevance to how anxiety disorders interfere with the ordinary activities of daily life and may also prove far more difficult to eradicate. We believe that conditioned suppression of baseline reward-seeking behavior offers an animal model for investigating the more pervasive consequences of anxiety disorders, which interfere with important activities in daily life—activities that are not necessary for survival but that nonetheless bring pleasure and fulfillment.

## Funding

This project was funded by grants from the National Institute of Mental Health (1R21MH086805 and 1R01MH091147 to MM).

### Conflict of interest statement

The authors declare that the research was conducted in the absence of any commercial or financial relationships that could be construed as a potential conflict of interest.
